# Solitary bone metastasis beneath the shoulder shield: coincidence or cause?

**DOI:** 10.3747/co.v13i4.98

**Published:** 2006-08

**Authors:** G. Fan, E. Sinclair, M. Christakis, L. Erhlich, J. Zubovits, E. Chow

**Keywords:** Solitary bone metastasis, recurrence, post-mastectomy radiotherapy

## Abstract

Post-mastectomy radiotherapy has been demonstrated to improve locoregional control in breast cancer patients. We report a case involving a 44-year-old breast cancer patient who presented with a solitary bone metastasis in the area beneath the shoulder shield, likely from a coincidental recurrence.

## 1. INTRODUCTION

Post-mastectomy radiotherapy has been demonstrated in randomized controlled trials to improve locoregional control in breast cancer patients 1,2. However, even after radiation treatment, 10%–30% of patients relapse locoregionally, 60%–70% relapse distally, and 10%–30% experience both types of relapse 3.

At our centre, when radiating the breast, it is standard practice to employ a shoulder shield to protect normal tissue. Here, we report a post-radiotherapy case of a solitary bone metastasis in the area beneath the shoulder shield in a breast cancer patient.

## 2. CASE HISTORY

**FIGURE 1:**
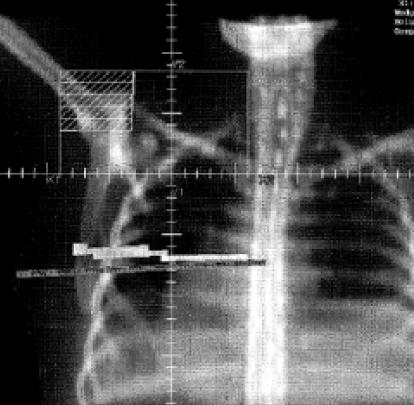
A shield was employed over the humeral head of the left shoulder during the radiation treatment.

A 44-year-old woman presented with a self-detected lump in her left breast. She underwent a left modified radical mastectomy, and pathology reviewed a 4.5-cm grade ii infiltrating ductal carcinoma with 9 of 16 lymph nodes involved. The tumour was progesterone-positive but estrogen- and her2/*neu*–negative. The metastatic work-up, including a bone scan, was negative. The patient was treated with dose-dense doxorubicin–cyclophosphamide/paclitaxel and with radiation to locoregional lymphatic-bearing areas in an adjuvant fashion. During the radiation treatment, a shield was employed to protect the patient’s left shoulder (Figure 1).

**FIGURE 2:**
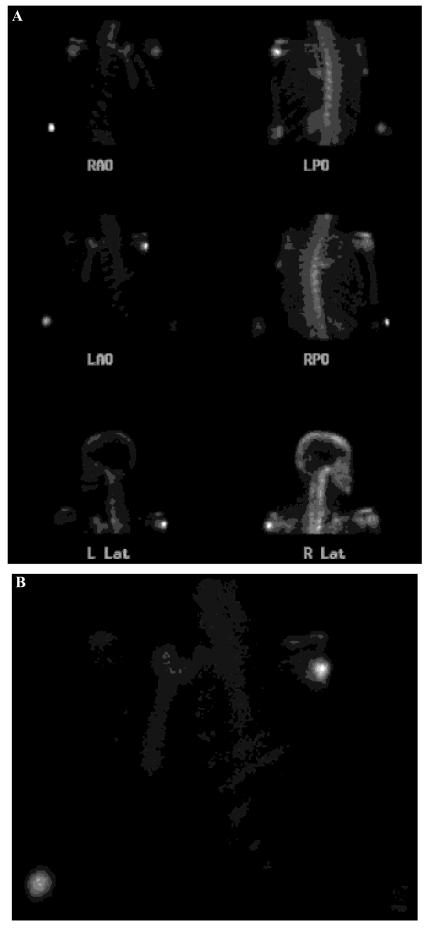
A technetium methylene diphosphonate bone scan showing increased tracer uptake in the posterior aspect of the left humeral head. (A) Left/right anterior/posterior oblique and lateral views. (B) Enlargement of left anterior oblique view.

**FIGURE 3:**
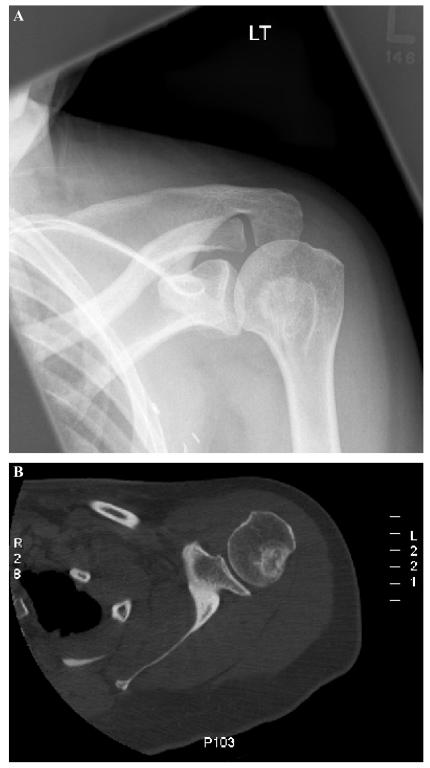
(A) Anterior/posterior radiograph of the left shoulder demonstrates a sclerotic lesion in the humeral neck corresponding to the abnormality on the bone scan. (B) Axial computed tomography image of the left shoulder confirms a predominantly sclerotic 1.9-cm × 1.8-cm × 2.0-cm eccentric lesion in the posterolateral humeral neck, in keeping with a metastatic focus.

A rheumatologist followed the patient because of ongoing benign sciatic pain. Because of increased pelvic pain, a bone scan and computed tomography (ct) scan were ordered. The imaging showed erosive changes in the right sacroiliac joint, associated with sclerosis of the adjacent bone—consistent with sacroiliitis. In addition, the bone scan revealed an increased uptake in the left shoulder (Figure 2). A ct scan and a radiograph of that area confirmed a 2.5-cm region of sclerosis within the left humeral head, representing bone metastasis (Figure 3).

**FIGURE 4:**
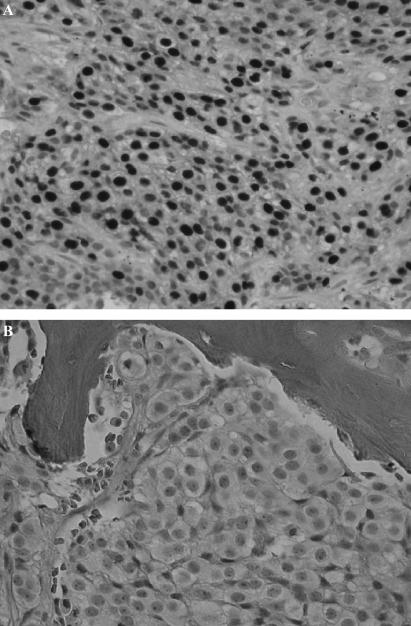
(A) Bone biopsy of the humerus shows large malignant epithelioid tumour cells growing in nests and sheets within spicules of bone. The appearance is consistent with a breast primary. (B) The tumour cells show strong staining for the progesterone receptor (consistent with a breast primary) and the same reactivity as the primary tumour.

Under ct guidance, a biopsy of the left humeral head was performed, and pathology confirmed meta-static carcinoma consistent with a breast primary (Figure 4). The patient had a repeat metastatic work-up, but showed no confirmed evidence of visceral involvement at the time of writing. She was treated with bisphosphonates and palliative radiotherapy for the bone metastasis in the left shoulder.

## 3. DISCUSSION AND CONCLUSIONS

This unfortunate woman was at risk of distant metastasis. At present, she has developed a solitary bone metastasis in the left shoulder corresponding to the shoulder shield in the radiation treatment.

Recurrence features in more than two thirds of breast cancer patients treated with post-mastectomy radiotherapy (3). Lee found that the greater the extent of axillary metastases, the higher the relapse rate. When axillary nodes are unremarkable, 18% of patients experience recurrence. Patients with 1–3 nodes and more than 4 nodes positive relapse at rates of 24% and 69% respectively (3). Bone is the main site of recurrence and accounts for 40%–60% of distant metastases (3).

Metastasis to the bone is common in breast cancer, arising in 20%–60% of patients and in up to 70%–85% at autopsy (4–7). More than 69% of these patients have a solitary recurrence confined to a single anatomic site, and 12% have multiple metastatic sites (8). However, only 3.5% of breast cancer patients develop long-bone metastases, and of those, 88% experience recurrence in the femur (9). When the humerus is involved, almost 90% of lesions arise in the proximal (42%) and diaphyseal (47%) regions (10). Metastases to the distal humerus are more infrequent, accounting for only 11% of cases (10).

The location of the metastasis in our patient was initially a curious surprise. Did the treatment protocol play a part in the area of metastasis? Or was it a coincidence that the bone lesion developed in the exact area shielded with the intent of protecting normal tissue? Most likely, the location of recurrence is a mere coincidence.
